# Pharmacokinetic evaluation of the PNC disassembler metarrestin in wild-type and Pdx1-Cre;LSL-Kras^G12D/+^;Tp53^R172H/+^ (KPC) mice, a genetically engineered model of pancreatic cancer

**DOI:** 10.1007/s00280-018-3699-0

**Published:** 2018-10-10

**Authors:** Tomas Vilimas, Amy Q. Wang, Samarjit Patnaik, Emma A. Hughes, Marc D. Singleton, Zachary Knotts, Dandan Li, Kevin Frankowski, Jerome J. Schlomer, Theresa M. Guerin, Stephanie Springer, Catherine Drennan, Christopher Dextras, Chen Wang, Debra Gilbert, Noel Southall, Marc Ferrer, Sui Huang, Serguei Kozlov, Juan Marugan, Xin Xu, Udo Rudloff

**Affiliations:** 1 Molecular Characterization Laboratory, Frederick National Laboratory for Cancer Research, Leidos Biomedical Research Inc., Frederick, MD 21702 USA; 2 Therapeutics for Rare and Neglected Diseases (TRND) Program, National Center for Advancing Translational Sciences, National Institutes of Health, 9800 Medical Center Drive, Rockville, MD 20850 USA; 3 Division of Preclinical Innovation, National Center for Advancing Translational Sciences, National Institutes of Health, 9800 Medical Center Drive, Rockville, MD 20850 USA; 4 Department of Bioengineering and Therapeutic Sciences, University of California San Francisco, San Francisco, CA 94158 USA; 5 Biophysics Graduate Group, University of California Berkeley, Berkeley, CA 94720 USA; 6 Rare Tumor Initiative (RTI), Pediatric Oncology Branch, Center for Cancer Research, National Cancer Institute, Hatfield Center, 10 Center Drive, 9000 Rockville Pike, Bethesda, MD 20892 USA; 7 Department of Medicinal Chemistry and Specialized Chemistry Center, University of Kansas, Lawrence, KS USA; 8 Center for Advanced Preclinical Research, Frederick National Laboratory for Cancer Research, Leidos Biomedical Research Inc., Frederick, MD 21702 USA; 9 Department of Cell and Molecular Biology, Northwestern University, Chicago, IL 60611 USA; 10 NIH Chemical Genomics Center, National Center for Advancing Translational Sciences, National Institutes of Health, 9800 Medical Center Drive, Bldg B, Rockville, MD 20850 USA

**Keywords:** Metarrestin, Peri-nucleolar compartment (PNC), KPC mice, Pharmacokinetics, Pharmacodynamics

## Abstract

**Purpose:**

Metarrestin is a first-in-class small molecule clinical candidate capable of disrupting the perinucleolar compartment, a subnuclear structure unique to metastatic cancer cells. This study aims to define the pharmacokinetic (PK) profile of metarrestin and the pharmacokinetic/pharmacodynamic relationship of metarrestin-regulated markers.

**Methods:**

PK studies included the administration of single or multiple dose of metarrestin at 3, 10, or 25 mg/kg via intravenous (IV) injection, gavage (PO) or with chow to wild-type C57BL/6 mice and KPC mice bearing autochthonous pancreatic tumors. Metarrestin concentrations were analyzed by UPLC–MS/MS. Pharmacodynamic assays included mRNA expression profiling by RNA-seq and qRT-PCR for KPC mice.

**Results:**

Metarrestin had a moderate plasma clearance of 48 mL/min/kg and a large volume of distribution of 17 L/kg at 3 mg/kg IV in C57BL/6 mice. The oral bioavailability after single-dose (SD) treatment was > 80%. In KPC mice treated with SD 25 mg/kg PO, plasma AUC_0–∞_ of 14400 ng h/mL, *C*_max_ of 810 ng/mL and half-life (*t*_1/2_) of 8.5 h were observed. At 24 h after SD of 25 mg/kg PO, the intratumor concentration of metarrestin was high with a mean value of 6.2 µg/g tissue (or 13 µM), well above the cell-based IC_50_ of 0.4 µM. At multiple dose (MD) 25 mg/kg/day PO in KPC mice, mean tissue/plasma AUC_0–24h_ ratio for tumor, spleen and liver was 37, 30 and 31, respectively. There was a good linear relationship of dosage to AUC_0–24h_ and C_24h_. AUC_0–24h_ MD to AUC_0–24h_ SD ratios ranged from two for liver to five for tumor indicating additional accumulation in tumors. Dose-dependent normalization of *FOXA1* and *FOXO6* mRNA expression was observed in KPC tumors.

**Conclusions:**

Metarrestin is an effective therapeutic candidate with a favorable PK profile achieving excellent intratumor tissue levels in a disease with known poor drug delivery.

**Electronic supplementary material:**

The online version of this article (10.1007/s00280-018-3699-0) contains supplementary material, which is available to authorized users.

## Introduction

Despite metastatic disease dissemination being the main cause of cancer-related mortality, there is a disproportionate paucity of successful drug development efforts selectively targeting metastasis [1–3]. Although there are various well characterized key processes and signal transduction pathways involved in metastatic colonization such as transcriptional programs induced by snail family transcriptional repressor 1 (SNAI1), inhibitor of DNA binding 1 (ID1), GATA-binding protein (GATA3), EMT, upregulated autocrine interleukin-6 (IL-6)-signal transducer and activator of transcription 3 (STAT3) signaling, or dysregulation of exosome formation and expression of various non-coding RNAs (reviewed in Steeg), these perturbations are either difficult to target, have redundant parallel signaling routes, are essential for normal tissue homeostasis, or are not selective for the evolved, metastatic disease stage [4].

**Fig. 1:**
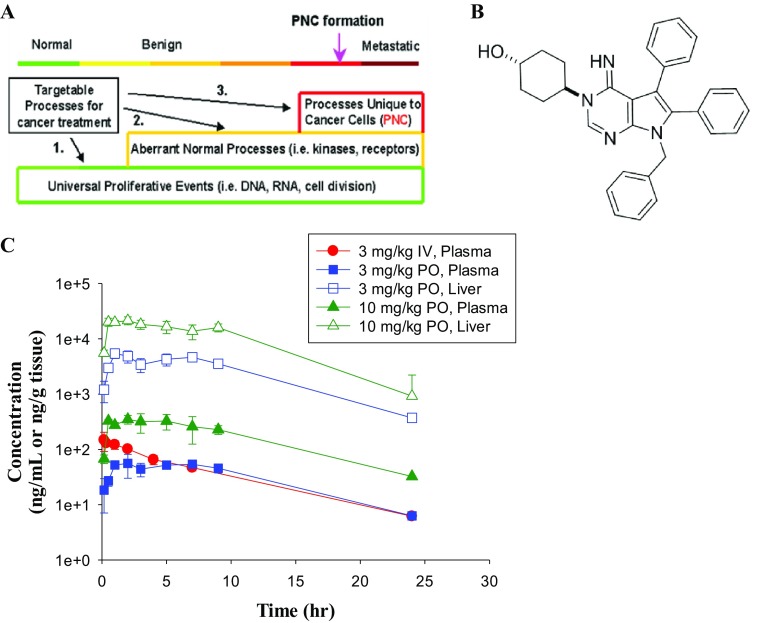
PNC disassembly as anti-metastasis therapy. **a** The PNC, not present in normal and pre-invasive disease stages, reflects the malignant phenotype at the late stage of the malignant transformation process, and thus can be a marker to select drugs that target processes selective for metastasis. **b** Chemical structure of metarrestin (ML-246). **c** Mean (± S.D.) metarrestin concentration–time profiles in C57BL/6 mice after single IV and PO administration (*n* = 3 per time point)

The perinucleolar compartment (PNC) is a small sub-nuclear organelle located adjacent to the nucleolus [5–7]. The PNC is enriched with RNA polymerase III transcripts, small nuclear RNAs, and RNA binding proteins [7–9]. Importantly, the PNC is unique to tumor cells from solid organ cancers [10]. PNC prevalence (the presence of one or more PNC structures per cell) correlates in various in vitro and in vivo cancer models, as well as in patient-derived clinical samples with the evolvement of malignant progression [10]. In correlative tissue studies of clinical samples from patients with breast, colon, and ovarian cancer PNC prevalence is both a strong predictive and prognostic marker of overall patients clinical outcome including overall survival [7, 11, 12]. Thus, the PNC is an attractive marker for drug development efforts targeting the metastatic disease state (Fig. 1a).

Metarrestin [*Trans*-4-(7-benzyl-4-imino-5,6-diphenyl-4,7-dihydro-3H-pyrrolo[2,3-d]pyrimidin-3-yl] cyclohexanol; Fig. 1b, MW: 474.6) was discovered from a high content drug screen of a > 140,000 compound library by selecting compounds via their ability to disassemble fluorescence-labelled PNC structures measured by high-throughput immunofluorescence microscopy imaging [13, 14]. To uncouple the PNC disassembling activity from the cytotoxic mode of action of DNA intercalators and topoisomerase I and II inhibitors previously known to disassemble the PNC, hits of the primary screen were re-screened, and excluded, via assays measuring cytotoxicity, apoptosis, or DNA intercalation displacement assays to arrive, after medicinal chemistry optimization, at metarrestin (ML-246) [14]. Metarrestin disassembles PNCs at submicromolar concentrations in solid organ cancer cell lines, suppresses metastasis formation, and extends survival in preclinical xenotransplantation models [15]. No toxicities have been observed at efficacious doses ranging between 5 and 25 mg/kg in mice [14, 15]. Thermostability and siRNA phenocopy studies have identified isoform 2 of the translation elongation factor eEF1A (eEF1A2) as a putative molecular target [15]. eEF1A2 is upregulated in pancreas cancer and other solid organ cancers compared to normal tissues, is overexpressed in metastatic disease sites (lymph nodes, distant organ metastasis) compared to primary tumors, and elevated expression levels are associated with poor survival outcome [16–20]. eEF1A2 is involved in many essential processes of the cell regulation including protein synthesis, maintenance of cytoskeletal architecture, or nuclear export regulation [21–24]. While some pro-oncogenic perturbations like upregulation of metalloproteinases governed by eEF1A2 or reduction of oxidative stress have been described, the exact mechanism of action as a bone fide cancer gene remains to be identified [25, 26].

To date, no comprehensive plasma, tissue, and tumor PK studies of this first-in-class compound have been reported so far. Here, we chose the Ras-driven, autochthonous KPC model to study PK and PK/PD relationships of metarrestin in pancreas cancer, a murine tumor model that recapitulates the challenges of poor drug delivery, a consequence of dense desmoplastic stroma and poor vascularization, observed in human pancreatic cancers [27–30]. The unique microenvironment in pancreatic cancer is well recognized to impede intratumoral drug penetration via perfusion and the inability to deliver efficacious concentrations to tumor cells limits efficacy of systemic chemotherapies. We correlated intratumor metarrestin exposure levels with administered dose levels, as well as markers impacted by metarrestin identified via differential gene expression and gene response patterns in KPC tumors vs matched normal, uninvolved pancreata which do not harbor PNCs. The knowledge of metarrestin PK properties at different exposure levels and the identification of dynamic biomarkers correlated with metarrestin exposure levels may drive the design of future relevant preclinical efficacy and safety studies, as well as early clinical development of metarrestin.

## Methods

### Chemicals

Metarrestin [ML-246; *Trans*-4-(7-benzyl-4-imino-5,6-diphenyl-4,7-dihydro-3H-pyrrolo[2,3-d]pyrimidin-3-yl) cyclohexanol] was manufactured in-house using the previously described 4-step synthesis sequence [14]. Purity (> 98%) was determined by LC-MS/MS and 1H-NMR analysis as previously reported [14]. To enhance bioavailability, the drug was micronized to particles of uniform small size with greater surface area using a technique proprietary to Crititech Inc. (Lawrence, Kansas) [31]. In brief, the drug substance was dissolved in organic solvent dichloromethane and precipitated using compressed super critical carbon dioxide as the anti-solvent. The produced material had 90% particles with a diameter less than 1.131 µm with a mean diameter of 0.829 µm (Suppl. Figure 1). The technique of attaining homogeneous particle size distribution by rapid change in solvent followed previous precedents to improve exposure in rodents [32–34]. Micronized metarrestin was used to formulate metarrestin-containing chow in NIH Haslan 31 rodent diet at 70 ppm or 170 ppm as equivalents of 10 mg/kg and 25 mg/kg daily doses based on 15–18% of life weight daily food consumption.

### Cell lines

Murine pancreatic KPC cancer cell lines were a kind gift of Ken Olive (Columbia University), and human pancreatic cancer lines FA-6 and PK-59 were generously provided by Nick Lemoine (Barts Cancer Institute, London, UK) and the RIKEN BRC cell bank (cellbank@brc.riken.jp). All cell lines were grown in RPMI supplemented with 10% fetal bovine serum (HyClone™, FisherScientific, Hampton, NH), 2 mM glutamine (Gibco Thermo Fisher, Waltham, MA), and 100 U penicillin/0.1 mg/mL streptomycin (Gibco) under 5% CO_2_.

### Animals

6–8-week old male C57BL/6 mice were purchased from Mouse Repository, Frederick National Laboratories for Cancer Research (FNLCR, Frederick, MD) or Charles River Laboratory (Frederick, MD). *KrasLSL-G12D*/+;*Trp53LSL-R172H*/+;*Pdx-1-Cre* (KPC) mice were bred as described previously [27]. All animal protocols were reviewed and approved by the Institute of Animal Care and Use Committee (IACUC) of their respective institutes, and studies were conducted in compliance with institutional animal use guidelines.

KPC mice were enrolled into PK studies when palpation followed by ultrasound examination within 24 h revealed 1 or 2 tumors with a combined volume of 100–250 mm^3^. Animals with three or more tumors per organ, pancreatic cysts or cystic tumors, signs of intestinal, pancreatic or gall bladder duct occlusions, or otherwise moribund/showing signs of dilapidation were excluded. Mice were randomized into treatment groups using their tumor volume and location inside pancreas, animal age, gender, and weight by an unbiased covariate randomization.

### Pharmacokinetic and pharmacodynamic studies in mice

For pharmacokinetic studies, metarrestin solutions prepared in 30% PEG-400 and 70% (20% w:v HP-β-CD in water) solution were administered to C57BL/6 mice at 3 mg/kg IV via tail vein injection, or 3 and 10 mg/kg orally via gavage. KPC mice were additionally treated with 25 mg/kg PO via gavage. The dosing volume was 3 mL/kg for IV route and 10 mL/kg for PO route. Dosing solutions were prepared on the day of administration. Blood samples were collected at 10, 20 min, 1, 2, 4, 7 and 24 h after 3 mg/kg IV administration. After oral administration of 3 and 10 mg/kg in C57BL6 mice, blood and liver samples were collected at 10 min, 0.5, 1, 2, 3, 5, 7, 9 and 24 h post dosing (*n* = 3 per time point). In KPC mice, blood, pancreatic tumor, spleen and liver samples were collected at 1, 2, 3, 6, 9, 12 and 24 h after 25 mg/kg PO administration.

For the assessment of multiple dose (MD) pharmacokinetics, KPC mice were treated with daily PO administration of 25 mg/kg metarrestin for 14 days. Plasma (100–300 µL), pancreatic tumors, spleen and liver samples were collected at 0, 3, 6, 9, 12, 15, 24 and 48 h after last dose from three mice at each time point. Daily dosing was carried for 14 days, with the last dose administered at 9:00 a.m. on Day 14. To assess the steady-state concentration of metarrestin in KPC mice, animals were treated with drug-formulated chow for 14 days. To control for daily cycle changes in feeding behavior, 9:00 a.m. on day 0 was designated as the study start time, and 9:00 a.m. on Day 14 was designated as the 0 h time point for sample collection. Blood, pancreatic tumor, spleen and liver tissue were collected at 0, 3, 6, 9, 12, 15, and 24 h from three mice at each time point while the animals were continuously fed with metarrestin-formulated chow on Day 14. Additional controls included mice with a matched drug-free diet, with blood and tissue samples collected at the initial (*t* = 0) and two intermediate (*t* = 9 h, *t* = 24 h) time points.

Blood samples were collected in K2EDTA tubes and plasma was harvested after centrifugation at 3000 rpm for 10 min. Tissue samples were weighed and flash-frozen for PK and PD mRNA expression analyses, embedded into OCT blocks for PNC determination and processed into FFPE blocks for histopathology assessment. All PK samples were stored at − 80 °C until analysis.

### Quantitative metarrestin analysis: UPLC-MS/MS

An ultra-performance liquid chromatography–tandem mass spectrometry (UPLC–MS/MS) method was developed to determine metarrestin concentrations in plasma and tissue samples. Mass spectrometric analysis was performed on a Waters Xevo TQ-S triple quadrupole instrument using electrospray ionization in positive mode. The separation was performed on an Acquity BEH C18 column (50 × 2.1 mm, 1.7 µ) using a Waters Acquity UPLC system with 0.6 mL/min flow rate and gradient elution. Mobile phase A was 0.1% formic acid in water, and B was 0.1% formic acid in acetonitrile (ACN). The column temperature was maintained at 60 °C. The calibration standards and quality control (QC) samples were prepared in the control blank mouse plasma and tissue homogenate. Tissues were homogenized with 3 or 6 volumes (v/w) of deionized water on the day of analysis. Proteins were precipitated from 10 µL plasma or 5 µL tissue homogenate with 200 µL ACN containing 100 ng/mL Tolbutamide as internal standard in 96-well plates. The mixture was vortexed for 2 min and centrifuged at 4000 rpm for 30 min at 4 °C. An aliquot of 150 µL supernatant was transferred to a 350-µL 96-well plate. 0.2 µL supernatant was injected for the UPLC–MS/MS analysis. Data were analyzed using MassLynx V4.1.

### Lower limit of quantitation (LLOQ), accuracy and precision

Bioanalytical method validation was performed in mouse plasma and mouse liver. The linear calibration range for metarrestin was 1.0–1000 ng/mL for plasma and 5.0–20,000 ng/g for liver homogenate. The lower limit of quantitation of the assay was 1.0 ng/mL in plasma and 5.0 ng/g in liver homogenate. The accuracy of calibration standards was between within 8% of the nominal values. Intra-assay variance and deviation from nominal values were calculated at low quality-control (LOQ, 1.0 ng/mL plasma and 5.0 ng/g liver), middle quality-control (MQC, 10.0 ng/mL and 500 ng/g), and high quality-control (HQC, 1000 ng/mL and 20,000 ng/g) levels for six replicates, each of the same analytical run (Suppl. Tables 1, 2). The variance of the QC samples (CV%, *n* = 3) ranged from 1.0 to 5.1% and the deviation was between 3 and 6% from nominal values. No endogenous interference was found at the retention time for metarrestin and internal standard as verified by testing control blank plasma and liver homogenate. Metarrestin was stable in mouse plasma after three freeze-thaw cycles.

### Pharmacokinetics calculation

The pharmacokinetic parameters were calculated using the non-compartmental approach (Models 200 and 201) of the pharmacokinetic software Phoenix WinNonlin, version 6.4 (Certara, St. Louis, MO). The area under the plasma concentration vs time curve (AUC) was calculated using the linear trapezoidal method. The slope of the apparent terminal phase was estimated by log linear regression using at least three data points and the terminal rate constant (*λ*) was derived from the slope. AUC_0–∞_ was estimated as the sum of the AUC_0–*t*_ (where *t* is the time of the last measurable concentration) and Ct/*λ*. The apparent terminal half-life (*t*_½_) was calculated as 0.693/*λ*.

### BODIPY®-metarrestin microscopy

The PNC disassembly assay has been previously described. For the detection of intracellular metarrestin, PC3M cells were cultured overnight at 37 °C in 5% CO_2_ in 384-well black clear bottom low-base plates (Cat# 4518; Corning, NY) at the density of 3000 cells/well in 30 µL/well DMEM supplemented with 10% FBS and 1% penicillin/streptomycin. 30 µL of a BODIPY^®^-labeled analog metarrestin, that was able to disassemble PNC in PC3M (PTB-GFP) cells expressing green fluorescent protein (GFP)-tagged polypyrimidine tract-binding protein 1 (PTB1) (described in [13]) at an IC_50_ 3.32 μM, was added at a final concentration of 5 µM metarrestin to each well and incubated at 37 °C in 5% CO_2_ for an additional 24 h. Cells were fixed with 4% for 10 min at room temperature. Fixed cells were washed with PBS containing 0.05% Tween-20 (PBS-T) for 30 min, incubated for 15 min at room temperature in 30 µL of 1 µg/mL Hoechst 33342 (Life Technologies, Carlsbad, CA) to visualize cell nuclei, then washed three times with PBS-T for 5 min per wash, and placed in 30 µL of PBS prior to microscopy. Confocal images were obtained using Opera Phoenix (PerkinElmer, Waltham, MA) and a 20× objective lens.

### Metarrestin metabolites

Hepatocytes monolayers from CD-1 mouse and SD rat, at a density of 1 million cells/mL, were incubated 10 µM metarrestin at 37 °C for 4 h under 5% CO_2_. Metabolic reactions were stopped by adding three volumes of acetonitrile (ACN) to the reaction mixture; the samples vortexed and centrifuged to precipitate the proteins. The supernatants were transferred to clean tubes for drying under a stream of nitrogen. The dried residues were reconstituted with 30% aqueous ACN and aliquots were injected onto LC/UV/MS system for metabolite ID and profiling. The LC/UV/MS system was Agilent 1100 HPLC (pumps, autosampler and PDA) interfaced to a Linear Trap Quadrupole (LTQ) ion trap mass spectrometer (ThermoFinnigan) with HPLC column: Luna C18(2) column, 250 × 2.0 mm, 5 µM. The HPLC mobile phase was a gradient between solvent A (0.05% TFA in water) and B (0.05% TFA in ACN) at a flow rate of 300 μL/min. At times 0, 2, 25, 28, 30, 31, 40 min the ratios of A:B were 95:5, 95:5, 35:65, 5:95, 5:95, 95:5, 95:5 respectively. The LTQ Mass Spectrometer ran a full scan for *m*/*z* 250–2000 and data dependent MS^n^ (*n* = 4) analysis.

### RNA-seq analysis

Pancreas cancer tissues samples or adjacent, uninvolved normal pancreas from vehicle or metarrestin-treated KPC animals collected at 0, 3, and 9 h after last dose of 25 mg/kg metarrestin were homogenized in PureLink Lysis Buffer (PureLink RNA Mini Kit, ThermoFisher Scientific Waltham, MA) at 4 °C using a TissueLyser II bead mill (Qiagen, Valencia, CA, manufactured by Retsch, Newtown, PA). Total RNA was isolated using the Life Technologies PureLink® RNA Mini Kit, quantified on a NanoDrop 1000 spectrophotometer (ThermoFisher Scientific, Waltham, MA). RNA Integrity Numbers (RIN) obtained on an Agilent 2100 Bioanalyzer (Agilent Technologies, Santa Clara, CA) ranged in tumor RNA preps from RIN 8.9 to 9.7 while non-tumor pancreas RIN ranged from 5.6 to 6.8.

Total RNA libraries were prepared using the Illumina TruSeq® Stranded Total RNA LT (with Ribo-Zero™Human/Mouse/Rat) kit according the manufacturer’s instructions. Double-stranded cDNA was purified using AMPure XP beads (Beckman Coulter Life Sciences, Indianapolis, IN), Sequencing was done using Illumina TruSeq V4 chemistry at 2 × 126 cycles on Illumina HiSeq2500. Image file processing to fastq output was done using Illumina HiSeq Real Time Analysis version RTA1.18.64 and Bcl2fastq2 version 2.17. Sequencing output ranged from 89 to 132 million reads per sample. Adapter trimming and base filtering was done using Trimmomatic software version 0.3, and the trimmed reads were aligned to mouse reference genome (GRCm38/mm10) and Ensembl annotation (version 70) using TopHat2 version 2.0.8. The list of identified differentially expressed genes was filtered by excluding transcripts with ≥ twofold change in metarrestin-treated vs untreated normal pancreas (to select targets with gene expression changes specifically in PNC-containing pancreatic tumors but not in PNC-lacking normal pancreas), and by preferentially selecting transcripts which showed ‘normalization’ of dysregulated gene expression levels in KPC tumors towards physiologic expression levels observed in normal pancreas using the following criteria: tumor treated with metarrestin (tm) vs tumor vehicle (tv) abs(fold change) ≥ 3, pancreas treated metarrestin (pm) vs pancreas vehicle (pv) absolute(foldChange) < 2, the product of the fold change of (tm vs tv) and (pv vs tv) was greater than 9.

### qRT-PCR

A total of 450 ng of total RNA was used for single-strand complementary DNA synthesis using a SuperScript III First-Strand kit (Invitrogen, Carlsbad, CA). Complementary DNA was amplified using the oligo dT20 (Invitrogen-Life Technologies) primer supplied in the kit. Reaction products were read in a Bio-Rad CFX96 Touch real-time PCR detection system (Bio-Rad Laboratories, Hercules, CA).

## Results

### Pharmacokinetics in wild-type and tumor-bearing KPC mice

#### PK in C57BL/6 mice after single IV and PO administration

Following single IV administration of 3 mg/kg in C57BL/6 mice, the plasma clearance of metarrestin was 48 mL/min/kg (Table 1). The steady-state volume of distribution (Vd_ss_) was 17 L/kg, indicating that the drug was distributed extensively in the body. This was confirmed by the high tissue concentrations, determined after oral administration (Table 1; Fig. 1). The terminal half-life (*t*_1/2_) was ~ 5 h. Following single oral administration of 3 and 10 mg/kg via gavage in C57BL/6, the absorption of the drug was very good with oral bioavailability > 80%. The maximum concentration (*C*_max_) in plasma, observed at 2 h after oral administration, was 55.7 ng/mL for the 3 mg/kg treatment and 349 ng/mL for the 10 mg/kg treatment, respectively. The corresponding AUC_0–∞_ values for 3 and 10 mg/kg treatment groups were 871 and 4790 ng h/mL. The *t*_1/2_ for the oral treatment was similar to that after IV treatment. Metarrestin concentrations in the liver were significantly higher than in the plasma, and the AUC ratio of liver/plasma was greater than 50 for both orally treated groups (Table 1).

**Table 1:** Metarrestin pharmacokinetics in C57BL/6 mice after single IV and PO administration

	Plasma	Plasma	Liver	Plasma	Liver
Dose (mg/kg)	3	3	3	10	10
Route	IV	PO	PO	PO	PO
AUC_0–24h_ (ng h/mL)	1010	823	66,100	4520	276,000
AUC_0–∞_ (ng h/mL)	1040	871	68,600	4790	281,000
*t*_1/2_ (h)	5	5.4	4.7	5.6	4.1
*T*_max_ (h)	NC	2	1	2	2
*C*_max_ (ng/mL)	NC	55.7	5420	349	21,400
CL_p_ (mL/min/kg)	48	NA	NA	NA	NA
Vd_ss_ (L/kg)	17	NA	NA	NA	NA
AUC_0–∞_ ratio (tissue/plasma)			79		59

*NC* Not calculated, *NA* not available

#### PK in KPC mice after single or multiple PO dose treatments

**Fig. 2:**
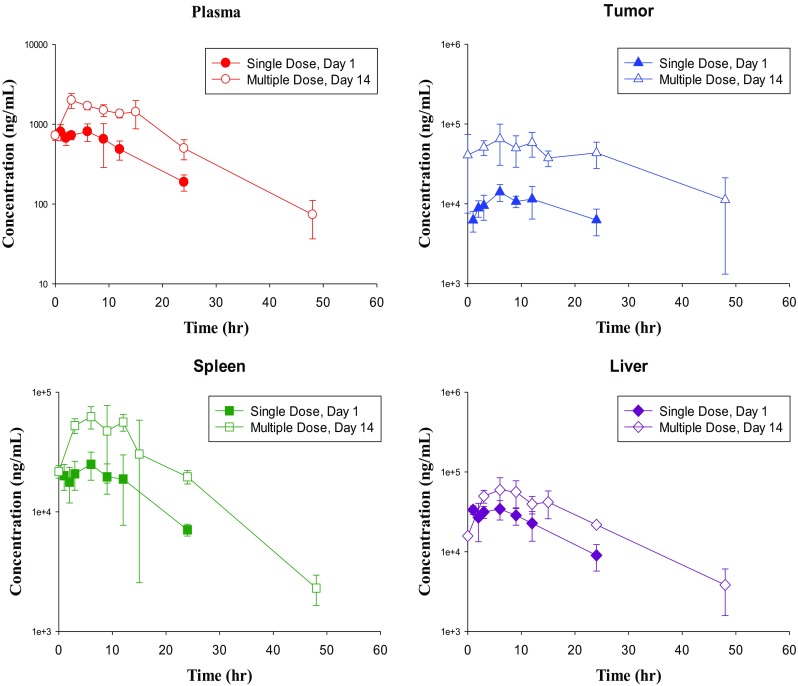
Days 1 and 14 mean (± S.D.) metarrestin concentration–time profiles in tumor-bearing KPC mice after single oral dose of 25 mg/kg or multiple oral doses of 25 mg/kg/day (*n* = 3 per time point)

**Fig. 3:**
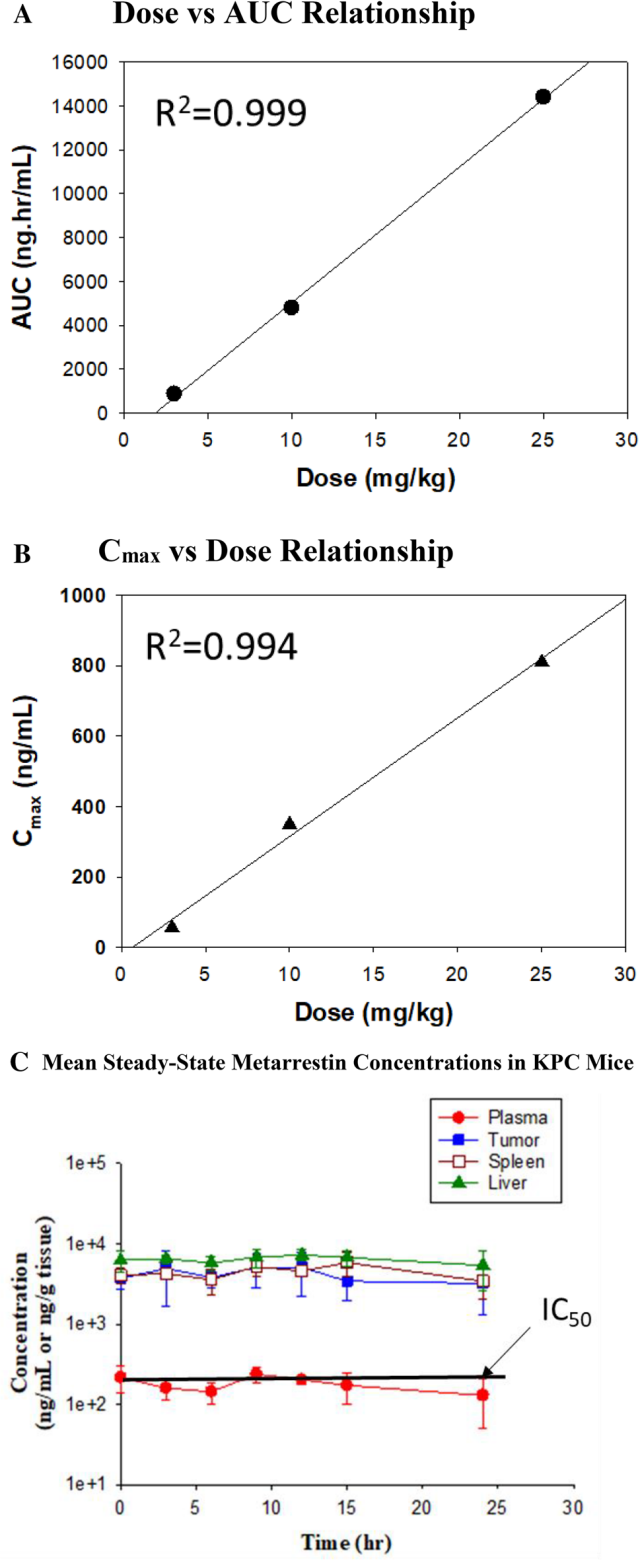
Administered dose levels (from 3 to 25 mg/kg) and plasma exposure of metarrestin are linearly correlated. **a** AUC_0–24h_, and **b** *C*_max_ vs dose plots for single dose PK studies in C57BL/6 and KPC mice (*R*^*2*^ regression coefficient). **c** Day 10 mean (± SD) metarrestin concentration–time profiles in tumor-bearing KPC mice after oral administration of metarrestin infused food chow (target dose 10 mg/kg/day; *n* = 3 per time point)

Following single oral administration of 25 mg/kg via gavage in KPC mice, *C*_max_ and AUC_0–∞_ in the plasma were 810 ng/mL and 14,400 ng h/mL, respectively (Table 2; Fig. 2). The *T*_max_ was observed at 6 h after drug administration, suggesting a prolonged absorption at a high dose of 25 mg/kg. The *t*_1/2_ of 8.5 h was also longer than 5–5.6 h observed at low doses of 3 and 10 mg/kg in C57BL/6 mice (Tables 1, 2). Nevertheless, in vivo systemic exposures (*C*_max_ and AUC_0–∞_) of metarrestin appeared to increase with the dose proportionally from 3 to 25 mg/kg (Fig. 3a, b) after single oral administration (3 and 10 mg/kg PO from C57BL/6 mice and 25 mg/kg PO from KPC mice).

**Table 2:** Metarrestin pharmacokinetics in tumor-bearing KPC mice after 25 mg/kg single or multiple dose treatment via oral gavage (PO)

Sample matrix	Single dose PK (single PO dose at 25 mg/kg)				PK on day 14 (multiple PO doses at 25 mg/kg/day)			
	Plasma	Tumor	Spleen	Liver	Plasma	Tumor	Spleen	Liver
AUC_0–24h_ (ng h/mL)	12,100	232,000	396,000	536,000	31,500	1,150,000	959,000	987,050
AUC_0–48h_ (ng h/mL)	NA	NA	NA	NA	38,400	1,810,000	1,220,000	1,290,000
AUC_0–∞_ (ng h/mL)	14,400	NC	495,000	653,000	39,300	2,090,000	1,250,000	1,350,000
*t*_1/2_ (h)	8.5	NC	9.7	9	8.2	18	8.2	9.6
*T*_max_ (h)	6	6	6	6	3	6	6	6
*C*_max_ (ng/mL)	810	14,000	25,000	34,200	2000	65,000	62,400	59,500
AUC_0–24h_ ratio (tissue/plasma)		19	33	44		37	30	31

*NC* Not calculated, *NA* not available

Similar to the initial observations in wild-type mice, metarrestin concentrations in KPC mice were higher in solid tissues than in plasma, and the AUC_0–24h_ ratio of tissue/plasma was 19 for tumor, 33 for spleen and 44 for liver after single administration of 25 mg/kg metarrestin by PO gavage (Table 2). The same high AUC tissue/plasma ratios were observed after multiple oral dosing with 25 mg/kg/day by oral gavage for 14 days (Table 2). On Day 14, the AUC_0–24h_ ratio of tissue/plasma was 37 for tumor, 30 for spleen and 31 for liver, respectively, after multiple administration of 25 mg/kg PO in KPC mice. Compared to Day 1 (data from SD Study), the mean metarrestin concentrations in tumor, spleen and liver were higher after MD treatment, suggesting drug accumulation in tissues after repeated dosing at this dose level (Table 2; Fig. 2). AUC_0–24h_ MD:AUC_0–24h_ SD ratio ranged from 1.8 for liver (987,000 ng h/g for MD:536,000 ng h/g for SD) to 5.0 for tumor (1,150,000 ng h/g for MD:232,000 ng h/g for SD) indicating accumulation with additional sequestration in tumors (Table 2). Daily dose metarrestin administration at 25 mg/kg by oral gavage for 14 days was not associated with any change in body weight, hypoactivity, or change in liver function tests compared to control animals treated with vehicle (Suppl. Table 3).

#### Steady-state intratumor PK in KPC mice

Intratumor metarrestin exposure levels were determined at steady-state with continuous administration of metarrestin. Previous anti-metastasis and survival studies in mice have identified a dose range of 10–25 mg/kg given either by PO gavage, or as drug-infused chow as dose levels associated with anti-tumor efficacy [15]. When dosed at 3 and 5 mg/kg PO, plasma concentrations of metarrestin measured lower than the IC_50_ values (~ 0.4 µM) determined in cell-based assays to effectively suppress PNC and achieve target inhibition. At 10 mg/kg PO single dose administration plasma concentrations remained above the IC_50_ for > 10 h. Thus, we chose a 10 mg/kg dose level which is sufficient to effectively suppress metastasis and extend survival [15]. KPC mice were fed with metarrestin-infused chow (70 ppm) for 10 days. On Day 10, plasma and tissues were collected from three mice every 3 h over a 24 h period while animals were maintained on the same metarrestin-containing diet. While metarrestin concentration in the plasma remained at or below the IC_50_, intratumor concentration exceeded the cell-based IC_50_ by ~ tenfold (Fig. 3c; Suppl. Table 4). The mean concentration (*C*_mean_; calculated by AUC_0–24h_/24 h) was 0.2 µM for plasma, 4.0 µM for tumor, 4.5 µM for spleen and 6.4 µM for liver, respectively. The tissue/plasma ratio was > 20 for all three tissues (Suppl. Table 5). Thus, intratumor concentration sufficient to achieve target inhibition, metastasis suppression and therapeutic response (prolonged survival) can be obtained using a solid formulation of metarrestin. It is also noticed that while *C*_max_ is generally lower when the drug is administrated in food chow compared to that after oral gavage, the plasma AUC_0–24h_ at steady-state from the metarrestin-chow treated PNC mice appears to be comparable to that when the drug is administered via single oral gavage at the same dose (4160 ng h/mL from steady-state chow study vs 4520 ng h/mL from single PO study at 10 mg/kg).

### Tumor pharmacokinetic/pharmacodynamic relationship

**Fig. 4:**
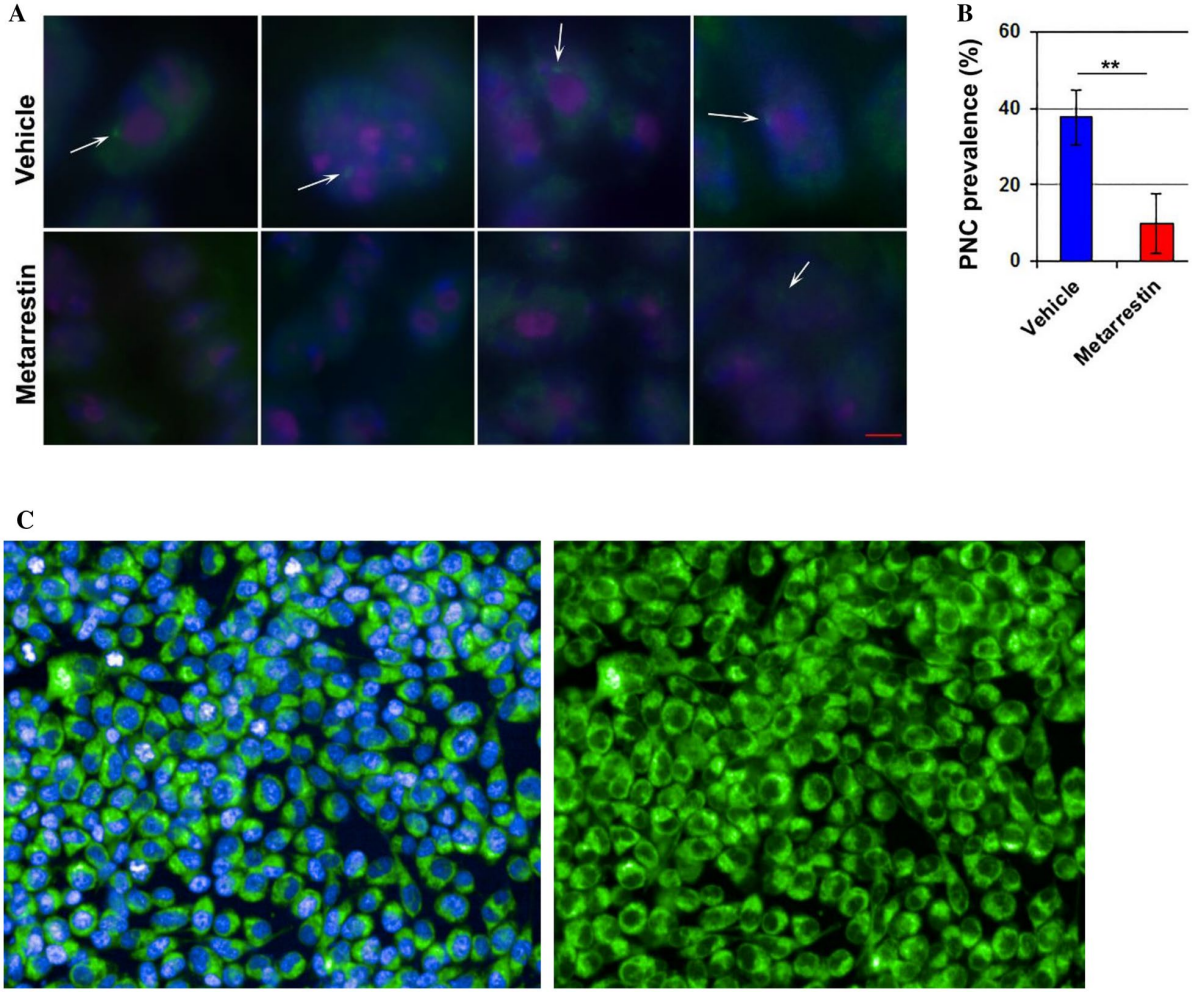

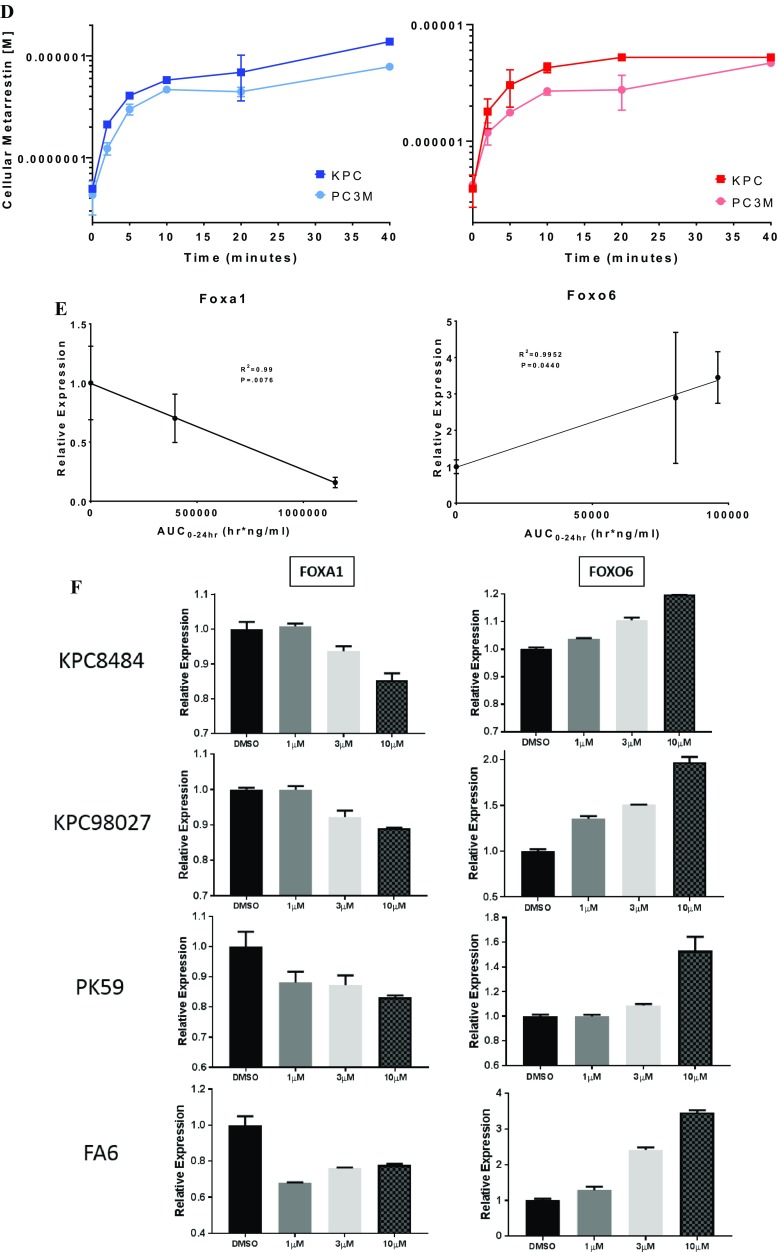
Intratumor metarrestin exposure correlates with PNC prevalence and mRNA expression levels of transcriptional regulators *FOXA1* and *FOXO6*. **a** Metarrestin disassembles the peri-nucleolar compartment (PNC) in KPC tumors. PNC structures in murine pancreas cancer KPC tumors visualized by immunofluorescence with α-PTB1 antibodies in tumors from vehicle-treated KPC mice (top panels) and from mice treated with 25 mg/kg metarrestin by PO gavage for 14 days (bottom panels). Representative images of individual tumors are shown; scale bar = 1 µm. **b** Quantification of PNC prevalence in 100 tumor cells in confirmed tumor areas of four tumors per treatment (*p < 0.05, **p < 0.01, ***p < 0.001; 2-tailed student’s *t* test). **c** 20× confocal images of PC3M cells following a 24-h incubation with 5 µM BODIPY®-labeled analog of metarrestin. Blue: Hoechst/Green: BODIPY®. **d** UPLC–MS/MS measurement of cellular metarrestin concentration in P3CM and KPC cells after treatment with 1 µM metarrestin (blue; left) and 10 µM (red; right) (in triplicates; error bars = S.D.; *x*-axis depicts time in min). **e** Correlation of expression levels of FOXA1 and FOXO6 with intratumor metarrestin exposure derived from different KPC cohorts. Relative expression levels of FOXA1 and FOXO6 determined by qRT-PCR and normalized to reference genes. FOXA1: mice dosed with vehicle, 25 mg/kg PO chow MD (AUC_0–24h_ 396,000 h ng/mL), and 25 mg/kg PO gavage MD (AUC_0–24h_ 1,150,000 h ng/mL). FOXO6: mice dosed with vehicle, 10 mg/kg PO chow (AUC_0–24h_ 80,600 h ng/mL) and 10 mg/kg PO steady-state chow (AUC_0–24h_ 96,100 h ng/mL). *N* ≥ 4 tumors per group, 2-tailed *R*^2^ correlation coefficient test. **f** qRT-PCR of FOXA1 and FOXO6 in murine and human pancreas cancer cells (see “Methods”) exposed for 24 h to increasing concentrations of metarrestin (*n* ≥ 2, in triplicates)

To establish a dose–response relationship between intratumor exposure levels of metarrestin and mechanism of action of the drug in vivo, we first confirmed the ability of metarrestin to suppress PNC structures in murine pancreatic KPC tumors. The prevalence of PNC bodies (expressed as the fraction of cells containing one or more PNCs) was 38.2% in untreated murine KPC tumors (Fig. 4a) which was similar to the PNC prevalence in the previously examined NOD/IL2 gamma (null) PANC1 metastasis model (35.1%) and higher than in cancer cell lines derived from primary pancreatic tumors (average ~ 23.5%) [15]. After 14-day treatment with 25 mg/kg metarrestin by oral gavage, PNC prevalence in KPC tumors was reduced to 9.8% (Fig. 4b).

To examine whether measured intratumor metarrestin concentrations measured in PK studies track with intracellular metarrestin concentration, we first decided to synthesize a fluorophore conjugated analog of metarrestin. Structure activity relationship studies around the structure of metarrestin were conducted in a high throughput assay that measured in dose dependence the ability of compounds to reducing PNC prevalence in PC3M cells [13, 14]. This medicinal chemistry effort produced an analog NCGC00244845 (PNC IC_50_ = 0.089 μM) that was more potent than metarrestin (PNC IC_50_ = 0.397 μM). This analog was chemically conjugated to the fluorophore boron-dipyrromethene (BODIPY®) to track the disassembly of the PNC in PC3M cancer cells by confocal microscopy (Suppl. Figure 2). The BODIPY-labelled analog of metarrestin (NCGC00387350) was able to disassemble PNC with IC_50_ = 3.317 μM in PC3M cells. Confocal microscopy showed homogenous intracellular accumulation of the drug in the cytosol (Fig. 4c). Next, to examine cellular metarrestin levels in relation to extracellular concentrations, we exposed KPC and PC3M cells at 1 and 10 µM concentrations of metarrestin and harvested cells at different timepoints. Consistent with the lipophilic nature of metarrestin, cellular levels of metarrestin reached 40–100% of media concentrations within 40 min (Fig. 4d). The homogeneous intracellular distribution of labeled metarrestin in cancerous cells, together with its ability to disassemble the PNC and a rapid equilibrium between media concentrations and cellular metarrestin levels in cancer cells harvested after short exposure to metarrestin-containing media suggest that measured intratumoral metarrestin concentrations correlate with intracellular metarrestin levels effective to disassemble the PNC.

To investigate whether the metabolite(s) of metarrestin contribute to the activity of the drug, we incubated hepatocytes from CD1-mice and SD rats with metarrestin for 4 h and analyzed the intracellular (cell lysate) and extracellular (media supernatant) fraction by HLPC. Three metabolites, that appeared to be products of oxidation via MS/MS analysis, were detected at very low (< 5%) concentrations compared to the parent metarrestin (Suppl. Figure 3). The main metabolite with (*M* + *H*)^+^ = 473 in mouse plasma, after MS/MS analysis, was proposed to be the ketone derivative of metarrestin, derived via the oxidation of the secondary alcohol on metarrestin (Suppl. Figure 4). Keto-metarrestin was synthesized and shown in direct comparison to metarrestin to be equipotent to metarrestin with respect to PNC disassembly in PC3M cells (Suppl. Figures 5, 6). Both, the low abundance of any metarrestin metabolites, as well as the absence of any increased activity of the main metabolite appear to indicate that metabolite(s) are not responsible for much of the therapeutic action of parent metarrestin.

To arrive at markers driven by metarrestin exposure, we next subjected untreated and metarrestin-treated KPC tumors and matched normal pancreata to unbiased gene expression profiling using RNA-sEq. To hone in on differentially expressed genes selectively induced by metarrestin, we first ruled out increased cleaved caspase expression levels by immunohistochemistry in treated tumors and pancreata to exclude any measure of cytotoxic off-target effects by the drug observed at higher concentrations in cell-based assays (Suppl. Figure 7). From the 364 transcripts with ≥ threefold change in treated vs untreated KPC tumors, we used a selection algorithm (see Methods) to identify 18 transcripts that were significantly changed (*p* value < 0.001) specifically in metarrestin-treated KPC tumors (Suppl. Figure 8). Two of the transcripts, *FOXA1* (known for its upregulation and pro-metastatic action in pancreatic cancer) and the putative tumor suppressor *FOXO6*, [35–37] showed metarrestin exposure-dependent expression changes in KPC tumors (Fig. 4c) and in cultured mouse and human pancreatic cancer cell lines (Fig. 4d). Thus, *FOXA1* and *FOXO6* may provide leads for future predictive biomarker development, assist in determination of the minimally efficacious dose, and add additional insight into the mechanism of action of metarrestin.

## Discussion

In this study, we define the PK and PK/PD relationship of metarrestin (ML-246) in the plasma, healthy tissues, and pancreatic tumors of wild-type and KPC (transgenic Ras-driven Pdx1-Cre;LSL-Kras^G12D/+^;Tp53^R172H/+^ pancreas cancer model) mice. Metarrestin is a pyrrolo-pyrimidin derivative [*trans*-4-(7-benzyl-4-imino-5,6-diphenyl-4,7-dihydro-3H-pyrrolo[2,3-d]pyrimidin-3-yl) cyclohexanol] able to disassemble the peri-nucleolar complex (PNC) without geno- or cytotoxicity [13, 14]. It was developed under the premise that the PNC is highly correlated with the metastatic phenotype in solid organ cancers based on several series of well-annotated clinical specimens [7, 11]. The compound exerts a novel mechanism of action by targeting isoform 2 of the translation elongation factor eEF1A (eEF1A2), which is a putative oncogene that has been shown to be upregulated in pancreatic and other cancers with expression levels that are inversely correlated with clinical outcome [16–20]. While phenocopy experiments via silencing or overexpressing eEF1A2 have linked metarrestin’s molecular target to PNC prevalence and metastasis formation in vivo, the exact impact of metarrestin on the pleiotropic molecular functions of eEF1A2 is still to be elucidated [15, 21]. It is likely that at concentrations sufficient to disassemble the PNC, metarrestin is also affecting non-canonical functions of eEF1A2 such as regulation of proteolysis, nuclear transport, oxidative stress, or regulation of gene transcription [23, 38]. Here, we establish that disassembly of the PNC and therapeutic efficacy (suppression of metastasis and extension of survival in the NSG *luc*[GLP] Panc1 metastasis model) can be achieved at 10 mg/kg dosing with solid or liquid formulations of metarrestin. These dose levels achieve intratumor AUC levels of > 80,000 ng h/mL and translate into single-digit micromolar (µmol/kg) metarrestin concentrations in tumors (Fig. 3) maintained for over 24 h from dosing. Such intratumor drug levels are particularly desirable in pancreatic cancer where the dense desmoplastic stroma and abnormal vasculature impede drug delivery. For this study, we chose the well-validated autochthonous KPC model which has been shown to recapitulate the unique cytoarchitectural, vascular, and microenvironmental biology of human pancreas cancer better than heterotopic or orthotopic xenotransplantation models [28].

One of the initial findings on the pharmacokinetic behavior of metarrestin elucidated within this study is the large volume of distribution, moderate clearance, and accumulation of the drug upon multiple dosing. After 14-day daily dosing with 25 mg/kg by PO gavage, metarrestin exposure (AUC_0–24h_) was about twofold higher in plasma and liver and nearly fivefold higher in pancreatic tumors than in animals treated with a single dose. This PK profile is in line with several observations reported previously [14]. The high steady-state drug concentration in tumor tissue (about 10-fold higher than the in vitro IC_50_) and the relatively long half-life suggest that less than daily dosing might be sufficient for anti-tumor activity in future preclinical and clinical studies. The high bioavailability (≥ 80%) and the *T*_max_ of < 2 h in plasma and < 6 h in most tissues indicated that drug is well absorbed after oral administration. Metarrestin has high tissue penetration, with AUC_tissue_:AUC_plasma_ ratio well above ten for the majority of solid organs, including pancreatic tumors, suggesting that therapeutically efficacious concentrations can be achieved even in pancreatic cancers known to be refractory for drug penetration and delivery [39]. With regard to drug delivery challenges, which are a significant hurdle in the development of more effective therapies for pancreatic cancer, there are two noteworthy observations on the pharmacokinetic behavior of metarrestin. First, the tissue distribution of metarrestin was largely independent of blood perfusion, as drug levels in poorly-vascularized pancreatic tumors were as high or higher as in some highly perfused solid organs. This suggests that therapeutically effective doses could be achieved in tumors without excessive toxicity in highly perfused organs. Second, there appears to be a retention effect of metarrestin in tumors compared to other solid organs. It is tempting to speculate that the abundance of the translation elongation factor A2 (eEF1A2), the molecular target of metarrestin which can comprise up to 1% of the total protein in cancer cells, accounts for the prolonged half life and increased AUC measures of intratumoral metarrestin compared to other organs. The sequestering of anti-cancer drugs via binding to components overexpressed in pancreatic tumors to overcome the poor drug delivery has recently garnered significant interest in the field through the introduction of nab-paclitaxel in combination with gemcitabine into clinical practice; paclitaxel bound to albumin (nab-paclitaxel, Abraxane®) binds via its albumin component to secreted protein acidic and rich in cysteine (SPARC) a stromal matrix protein overexpressed in many pancreatic and breast cancers and intratumoral paclitaxel levels correlate with tumoral SPARC expression levels [40, 41].

Previous in vitro studies showed that the PNC is restored, the nucleolus is reorganized, and cell proliferation recovers after removal of metarrestin, suggesting that the drug acts via a cytostatic mechanism [14, 15]. Thus, it may be especially important to continuously maintain metarrestin exposure at a therapeutically effective level, but findings from this study and our previous report suggest that this can be readily achieved in tumors without detectable toxicity in healthy tissues [15]. Using RNA-seq gene expression profiling, we identified 18 transcripts as candidate metarrestin PD markers, which are restored to normal pancreas expression level after metarrestin treatment. These include the metastasis enhancer *FOXA1* and the putative tumor suppressor *FOXO6*. Interestingly, the known role of *FOXA1* in the metastatic transition of pancreas cancer, originally identified in the KPC animal model used in this study, and the tumor suppressor functions of *FOXO6* in lung cancer cells provide biologically plausible links to the anti-metastatic phenotype of metarrestin [35–37].

In summary, here we report a comprehensive PK profile of metarrestin in wild-type and tumor-bearing KPC mice. The PK properties described here, including high oral bioavailability, a large volume of distribution, relatively long half-life, and moderate clearance with accumulation in tumor tissues will inform the design of future preclinical and IND enabling studies. The excellent tissue penetration of metarrestin and the absence of detectable toxicity make metarrestin an attractive therapeutic candidate for future development, in particular, in tumor types with dense desmoplastic stroma and poor drug delivery.

## Electronic supplementary material

Below is the link to the electronic supplementary material.

Supplementary material 1 (DOCX 3972 KB)

## Abbreviations

ACN: Acetonitrile
AUC: Area under the curve
CL_p_: Plasma clearance
*C*_max_: Maximum (or peak) serum concentration
CV: Coefficient of variance
eEF1A: Eukaryotic translation elongation factor 1 alpha
EMT: Epithelial to mesenchymal transition
FOXA1: Forkhead box protein A1
FOXO6: Forkhead box O6
GFP: Green fluorescent protein (GFP)
HPLC: High-performance liquid chromatography
^1^H-NMR: Proton nuclear magnetic resonance
HP-β-CD: (2-Hydroxylpropyl)-beta-cyclodextrin
HQC: High quality-control
IC_50_: Inhibitory concentration 50
IND: Investigational new drug
IV: Intravenous
K2EDTA: Dipotassium ethylenediaminetetraacetic acid
LC–MS/MS: Liquid chromatography–tandem mass spectrometry
LLOQ: Lower limit of quantitation
LOQ: Low quality-control
LTQ: Linear trap quadrupole
MD: Multiple dose
MQC: Middle quality-control
NOD/IL2: Nonobese diabetic/interleukin-2
NSG: NOD scid gamma
OCT: Optimal cutting temperature compound
PD: Pharmacodynamic
PK: Pharmacokinetics
PNC: Perinucleolar compartment
PO: Per os
Ppm: Parts per million
PTB1: Polypyrimidine tract-binding protein 1
QC: Quality control
RIN: RNA integrity numbers
RPMI: Roswell Park Memorial Institute Media
S.D.: Standard deviation
SD: Single dose
SPARC: Secreted protein acidic and rich in cysteine
TFA: Trifluoroacetic acid
UPLC–MS/MS: Ultra-performance liquid chromatography–tandem mass spectrometry
Vd_ss_: Steady-state volume of distribution

## References

[1] Siegel RL, Miller KD, Jemal A (2016). Cancer statistics. CA Cancer J Clin.

[2] Tevaarwerk AJ (2013). Survival in patients with metastatic recurrent breast cancer after adjuvant chemotherapy: little evidence of improvement over the past 30years. Cancer.

[3] Bernards N (2013). No improvement in median survival for patients with metastatic gastric cancer despite increased use of chemotherapy. Ann Oncol.

[4] Steeg PS (2016). Targeting metastasis. Nat Rev Cancer.

[5] Pollock C, Huang S (2009). The perinucleolar compartment. J Cell Biochem.

[6] Norton JT (2009). The perinucleolar compartment is directly associated with DNA. J Biol Chem.

[7] Slusarczyk A (2010). Structure and function of the perinucleolar compartment in cancer cells. Cold Spring Harb Symp Quant Biol.

[8] Wang C (2003). RNA polymerase III transcripts and the PTB protein are essential for the integrity of the perinucleolar compartment. Mol Biol Cell.

[9] Pollock C (2011). Characterization of MRP RNA-protein interactions within the perinucleolar compartment. Mol Biol Cell.

[10] Norton JT, Huang S (2013). The perinucleolar compartment: RNA metabolism and cancer. Cancer Treat Res.

[11] Kamath RV (2005). Perinucleolar compartment prevalence has an independent prognostic value for breast cancer. Cancer Res.

[12] Norton JT (2008). Perinucleolar compartment prevalence is a phenotypic pancancer marker of malignancy. Cancer.

[13] Norton JT (2009). Automated high-content screening for compounds that disassemble the perinucleolar compartment. J Biomol Screen.

[14] Frankowski K et al (2010) Discovery and development of small molecules that reduce PNC prevalence. In: Probe reports from the NIH molecular libraries program, Bethesda (MD)

[15] Frankowski KJ (2018). Metarrestin, a potent perinucleolar compartment inhibitor, effectively suppresses metastasis. Sci Transl Med.

[16] Tomlinson VA (2005). Translation elongation factor eEF1A2 is a potential oncoprotein that is overexpressed in two-thirds of breast tumours. BMC Cancer.

[17] Tomlinson VA (2007). Expression of eEF1A2 is associated with clear cell histology in ovarian carcinomas: overexpression of the gene is not dependent on modifications at the EEF1A2 locus. Br J Cancer.

[18] Scaggiante B (2012). Dissecting the expression of EEF1A1/2 genes in human prostate cancer cells: the potential of EEF1A2 as a hallmark for prostate transformation and progression. Br J Cancer.

[19] Kawamura M (2014). The prognostic significance of eukaryotic elongation factor 1 alpha-2 in non-small cell lung cancer. Anticancer Res.

[20] Zang W (2015). miR-663 attenuates tumor growth and invasiveness by targeting eEF1A2 in pancreatic cancer. Mol Cancer.

[21] Gross SR, Kinzy TG (2005). Translation elongation factor 1A is essential for regulation of the actin cytoskeleton and cell morphology. Nat Struct Mol Biol.

[22] Shamovsky I (2006). RNA-mediated response to heat shock in mammalian cells. Nature.

[23] Mateyak MK, Kinzy TG (2010). eEF1A: thinking outside the ribosome. J Biol Chem.

[24] Huang HY, Hopper AK (2015). In vivo biochemical analyses reveal distinct roles of beta-importins and eEF1A in tRNA subcellular traffic. Genes Dev.

[25] Chuang SM (2005). Proteasome-mediated degradation of cotranslationally damaged proteins involves translation elongation factor 1A. Mol Cell Biol.

[26] Xu C, Hu DM, Zhu Q (2013). eEF1A2 promotes cell migration, invasion and metastasis in pancreatic cancer by upregulating MMP-9 expression through Akt activation. Clin Exp Metastasis.

[27] Hingorani SR (2005). Trp53R172H and KrasG12D cooperate to promote chromosomal instability and widely metastatic pancreatic ductal adenocarcinoma in mice. Cancer Cell.

[28] Olive KP (2009). Inhibition of Hedgehog signaling enhances delivery of chemotherapy in a mouse model of pancreatic cancer. Science.

[29] Feig C (2012). The pancreas cancer microenvironment. Clin Cancer Res.

[30] Provenzano PP (2012). Enzymatic targeting of the stroma ablates physical barriers to treatment of pancreatic ductal adenocarcinoma. Cancer Cell.

[31] Takeuchi H, Yamamoto H, Kawashima Y (2001). Mucoadhesive nanoparticulate systems for peptide drug delivery. Adv Drug Deliv Rev.

[32] Rasenack N, Muller BW (2002). Dissolution rate enhancement by in situ micronization of poorly water-soluble drugs. Pharm Res.

[33] Kerc J (1999). Micronization of drugs using supercritical carbon dioxide. Int J Pharm.

[34] Mutalik S (2008). Enhancement of dissolution rate and bioavailability of aceclofenac: a chitosan-based solvent change approach. Int J Pharm.

[35] Roe JS (2017). Enhancer reprogramming promotes pancreatic cancer metastasis. Cell.

[36] Hu HJ (2015). FoxO6 inhibits cell proliferation in lung carcinoma through up-regulation of USP7. Mol Med Rep.

[37] Wolf J (2014). An in vivo RNAi screen identifies SALL1 as a tumor suppressor in human breast cancer with a role in CDH1 regulation. Oncogene.

[38] Abbas W, Kumar A, Herbein G (2015). The eEF1A proteins: at the crossroads of oncogenesis, apoptosis, and viral infections. Front Oncol.

[39] LaBarge MA (2010). The difficulty of targeting cancer stem cell niches. Clin Cancer Res.

[40] Neesse A (2014). SPARC independent drug delivery and antitumour effects of nab-paclitaxel in genetically engineered mice. Gut.

[41] Von Hoff DD (2013). Increased survival in pancreatic cancer with nab-paclitaxel plus gemcitabine. N Engl J Med.

