# Supplementary material for: Pharmacokinetic evaluation of the PNC disassembler metarrestin in wild-type and Pdx1-Cre;LSL-KrasG12D/+;Tp53R172H/+ (KPC) mice, a genetically engineered model of pancreatic cancer
Source: Cancer Chemother Pharmacol. 2018 Oct 10;82(6):1067–80. doi: 10.1007/s00280-018-3699-0 (PMC6267684; doi:10.1007/s00280-018-3699-0)
Supplement: Supplementary file 1 — Supplementary material 1 (DOCX 3972 KB) [file 280_2018_3699_MOESM1_ESM.docx]

**Supplemental Figures**

**Suppl. Fig. 1**. Homogeneous particle size distribution after micronization via rapid change in solvent. Size distribution of metarrestin particles (differential distribution of particle size (in µm; x-axis) versus relative distribution (%; y-axis) by size exclusion chromatography) before (left) and after (right) particle size reduction.

**Suppl. Fig. 2**. Synthesis scheme to boron-dipyrromethene (BODIPY) labeled analog of metarrestin. Activity of analogs in PC3M cells.

NCGC00247785

Metarrestin

PNC IC_50_ = 0.397μM

NCGC00244845

Metarrestin analog

PNC IC_50_ = 0.089μM

NCGC00387350

BODIPY tagged metarrestin analog

PNC IC_50_ = 3.317μM

**Suppl. Fig. 3**. HPLC chromatogram (top) of hepatocytes incubated for 4 hours with metarrestin (NCG00247785). Extracted ions chromatogram (below) of peaks: 474 (parent) and metabolites 490a, 490b, 472.

**Suppl. Fig. 4**. Predicted metabolic pathways of metarrestin with MS/MS spectra of metabolites and structure elucidation.


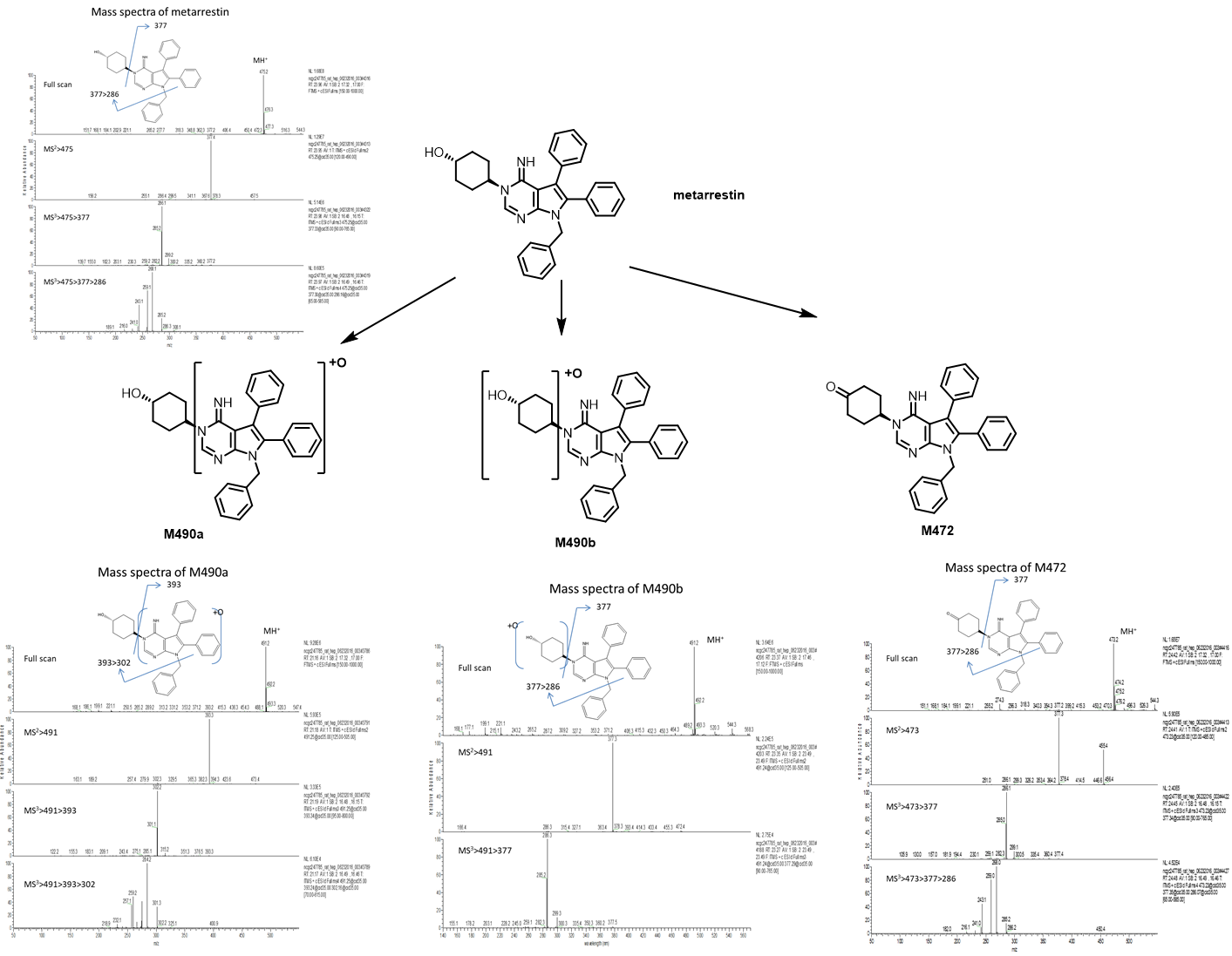


**Suppl. Fig. 5**. Synthesis and characterization of keto metarrestin. Metarrestin (200 mg, 0.421 mmol) was taken in DMSO (5 mL) and treated with Burgess Reagent (131mg, 0.548 mmol) under nitrogen according to the method described in literature. The reaction was stirred for 2 hours. Analysis via LCMS revealed very little ketone formation. The mixture was diluted with ethyl acetate and washed with water. The top ethyl acetate layer was dried with anhydrous magnesium sulfate which was filtered off. The filtrate was concentrated and subjected to purification by flash silica gel chromatography. This provided a fraction that was enriched with the ketone but still had metarrestin. A portion of this was loaded on a thin layer 20x20 cm glass plate with 1mm silica gel and eluted with 5-10% methanol in dichloromethane twice to provide a sample of the ketone derivative of metarrestin that was not contaminated with metarrestin (~7% yield).

^1^H NMR (400 MHz, Chloroform-d) δ 7.75 (s, 1H), 7.33 – 7.15 (m, 11H), 7.11 – 7.01 (m, 2H), 6.99 – 6.91 (m, 2H), 5.69 – 5.49 (m, 1H), 5.28 (s, 2H), 2.73 – 2.60 (m, 2H), 2.60 – 2.50 (m, 2H), 2.46 - 2.36 (m, 2H), 2.05 (qd, *J* = 12.8, 4.5 Hz, 2H).

LCMS of purified metarrestin ketone:


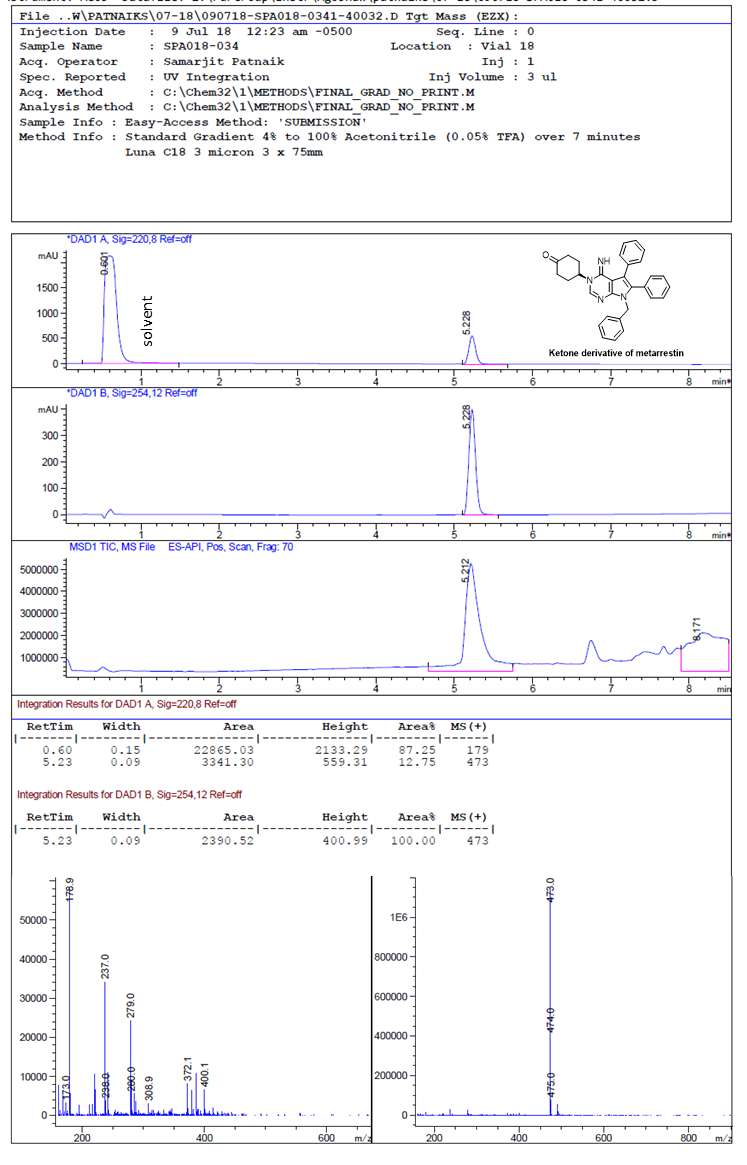


**Suppl. Fig. 6**. PNC disassembly of metarrestin (blue) vs keto-metarrestin (red) in P3CM cells (N=2 independent experiments; in triplicates; top). Representative images of PNC treated with vehicle (left) and at 10µM keto-metarrestin are shown on the bottom. Arrows (white) indicate peri-nucleolar compartment (PNC).


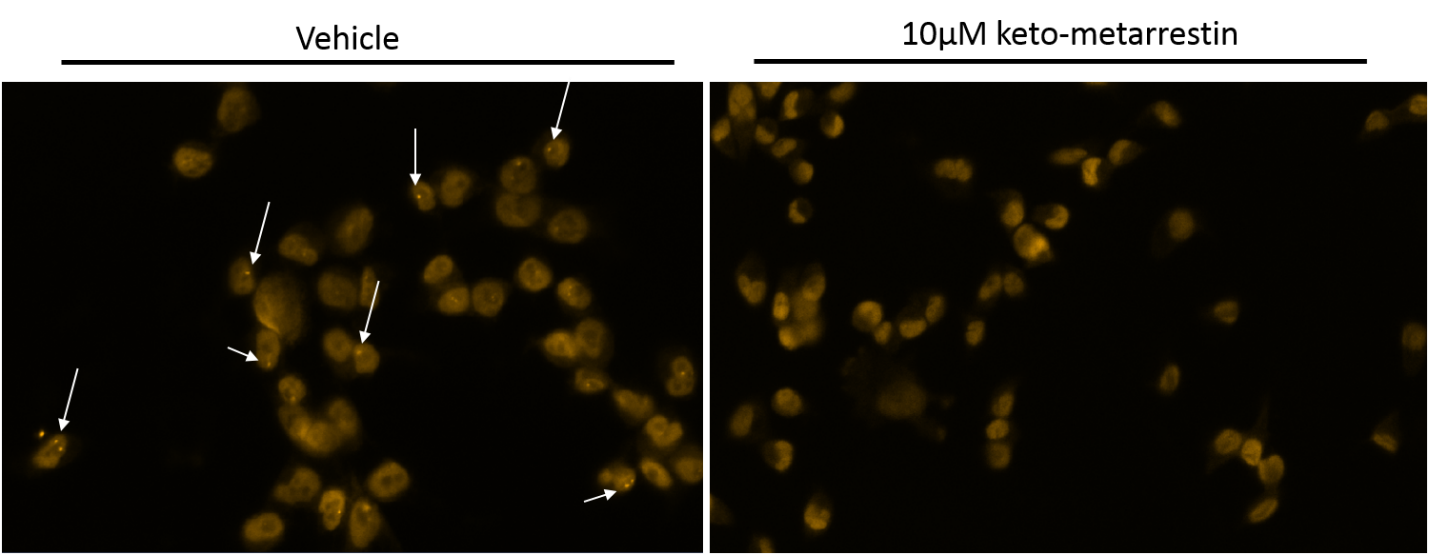


**Suppl. Fig. 7**. Metarrestin does not induce cell death in KPC tumors treated with 25mg/kg metarrestin by gavage (PO) for 14 days. Nuclear cleaved caspase 3 expression levels in metarrestin-treated KPC tumors. Representative images of cleaved caspase 3 immunohistochemistry in tumors of KPC mice treated with vehicle (top) or 25mg/kg metarrestin for 14 days (bottom). Images 20×, inset 40×.


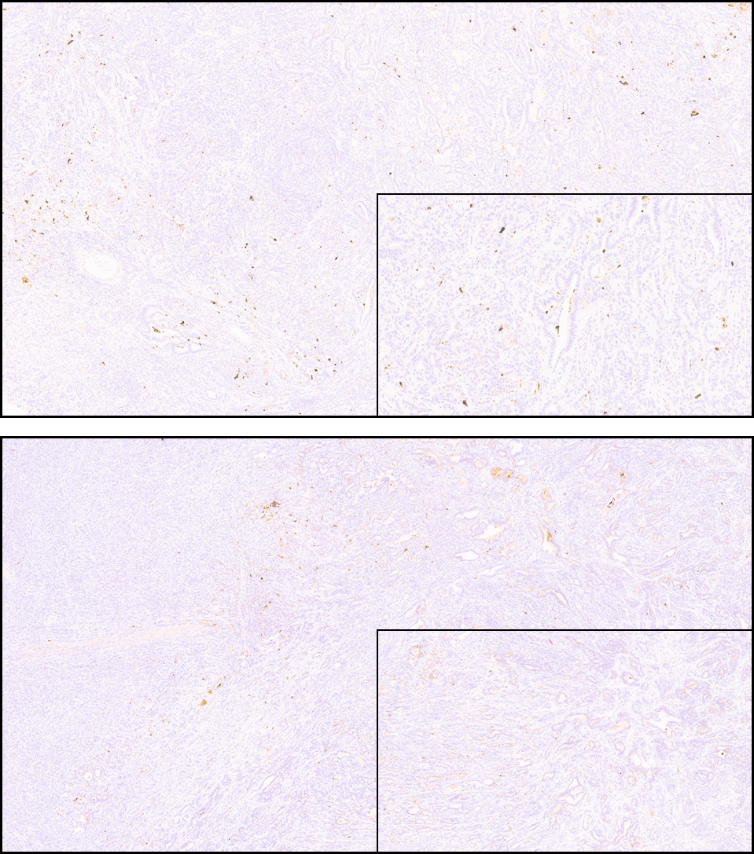


**Suppl. Fig. 8**. Relative mRNA expression of 18 genes with dysregulated expression in vehicle-treated KPC tumors and normalized expression (relative to non-tumor uninvolved pancreas) in KPC tumors treated with a 10mg/kg metarrestin diet, identified from RNA-seq gene expression profiling of KPC tumors.

**Supplemental Tables**

**Suppl. Table 1**. Validation of quantitative metarrestin analysis assay, mouse plasma. Intraday (n=6) and interday (n=18, three repeats) results. CV, coefficient of variance.

| QC Conc (ng/mL) | Intraday | | | Interday | | |
| --- | --- | --- | --- | --- | --- | --- |
|  | Conc (ng/mL) | CV (%) | Deviation from nominal values (%) | Conc (ng/mL) | CV (%) | Deviation from nominal values (%) |
| LOQ (1.00) | 1.05 | 2.3 | +5 | 0.956 | 9.0 | -4 |
| MQC (10.0) | 9.69 | 3.1 | -3 | 9.32 | 4.5 | -7 |
| HQC (1000) | 937 | 8.2 | -6 | 1050 | 2.9 | +5 |

**Suppl. Table 2**. Validation of quantitative metarrestin analysis assay, mouse liver tissue. Intraday (n=6) and interday (n=18, three repeats) results.

| QC Conc (ng/g) | Intraday | | | Interday | | |
| --- | --- | --- | --- | --- | --- | --- |
|  | Conc (ng/g) | CV (%) | Deviation from nominal values (%) | Conc (ng/g) | CV (%) | Deviation from nominal values (%) |
| LOQ (5.00) | 5.01 | 8.4 | 0 | 4.81 | 7.0 | +4 |
| MQC (500) | 513 | 3.3 | +3 | 510 | 8.1 | +2 |
| HQC (20000) | 19500 | 4.4 | -3 | 19600 | 4.1 | -2 |

**Suppl. Table 3**. Preliminary toxicity profile of 10 female and 10 male Balb/c mice treated with vehicle (Group 1) and 25mg/kg metarrestin by PO gavage (Group 2) for 14 days (K, potassium; CRE, creatinine; P04, phosphorus; ALP, alkaline phosphatase; ALT, alanine transaminase; AST, aspartate transaminase; LD, lactate dehydrogenase; TP, total protein; GLOB, globulin; CHOL, cholesterol).

| **Mortality and Clinical Observation** | | |
| --- | --- | --- |
| **Males** | **Group 1** | **Group 2** |
| **N=10** | **0 mg/kg** | **25 mg/kg** |
| Death | **0** | **0** |
| Hypoactivity | 0 | 0 |
| Rough coat | 0 | 6 |
| Cold to touch | 0 | 0 |
| Thin | 0 | 0 |
| Scant feces | 0 | 0 |
| **Females** | **Group 1** | **Group 2** |
| **N=10** | **0 mg/kg** | **25 mg/kg** |
| Death | **0** | **0** |
| Rough coat | 0 | 0 |
| Scant feces | 0 | 0 |
| **Weight** |  |  |
| **Males** | **Group 1** | **Group 2** |
| **N=10** | **0 mg/kg** | **25 mg/kg** |
| Day 1 | 31.1 ±1.73 | 31.4 ± 1.38 |
| Day 7 | 32.4 ± 2.01 | 33.3 ± 2.02 |
| Day 15 | 33.2 ± 2.46 | 34.4 ± 2.33 |
| **Females** | **Group 1** | **Group 2** |
| **N=10** | **0 mg/kg** | **25 mg/kg** |
| Day 1 | 23.2 ± 1.66 | 23.1 ± 1.55 |
| Day 7 | 24.1 ± 2.00 | 24.3 ± 1.32 |
| Day 15 | 25.7 ± 2.20 | 25.8 ± 1.70 |
| **Weight Change** | |  |
| **Males** | **Group 1** | **Group 2** |
| **N=10** | **0 mg/kg** | **25 mg/kg** |
| Day 1 to 4 | 1.0 ± 0.64 | 1.1 ± 0.90 |
| Day 4 to 7 | 0.3 ± 0.64 | 0.9 ± 0.59 |
| Day 1 to 15 | 2.1 ± 1.23 | 3.1 ± 1.51 |
| **Females** | **Group 1** | **Group 2** |
| **N=10** | **0 mg/kg** | **25 mg/kg** |
| Day 1 to 4 | 0.0 ± 0.88 | 0.0 ± 0.80 |
| Day 4 to 7 | 0.9 ± 0.92 | 1.2 ±0.67 |
| Day 1 to 15 | 2.5 ± 1.44 | 2.7 ± 1.04 |
| **Liver chemistry** | |  |
| **Males** | **Group 1** | **Group 2** |
|  | **0 mg/kg** | **25 mg/kg** |
| K | 7.3 ±0.28 | 7.3 ± 0.21 |
| CRE | 0.11 ± 0.018 | 0.09 ± 0.015* |
| P04 | 9.9 ± 1.09 | 10.2 ± 0.50 |
| ALP | 81 ± 12.3 | 84 ± 13.5 |
| ALT | 175 ±86.9 | 137 ± 67.4 |
| AST | 137 ± 67.4 | 88 ± 24.3 |
| LD | 172 ± 37.6 | 659 ± 154.6 |
| **Females** | **Group 1** | **Group 2** |
|  | **0 mg/kg** | **25 mg/kg** |
| TP | 5.2 ± 0.29 | 5.2 ± 0.21 |
| GLOB | 2.0 ± 0.18 | 2.1 ± 0.13 |
| ALP | 135 ± 33.9 | 136 ± 13.8 |
| CHOL | 125 ± 9.1 | 123 ± 18.4 |
| ALT | 77 ± 23.0 | 100 ± 47.6 |
| AST | 105 ± 30.4 | 100 ± 28.3 |
| LD | 591 ± 147.9 | 495 ± 89.7 |

**Suppl. Table 4.** Mean Day 10 metarrestin concentration in plasma and tumor at steady-state with 10 mg/kg/day (70 ppm) metarrestin chow dosing in tumor-bearing KPC mice.

| Time (hr) | 0 | 3 | 6 | 9 | 12 | 15 | 24 |
| --- | --- | --- | --- | --- | --- | --- | --- |
| Plasma concentration (μM) | 0.46 | 0.33 | 0.30 | 0.50 | 0.43 | 0.36 | 0.27 |
| Tumor concentration (μM) | 7.9 | 10.3 | 8.0 | 10.8 | 10.7 | 7.2 | 6.7 |

**Suppl. Table 5**. Metarrestin pharmacokinetics in tumor-bearing KPC mice on day 10 after multiple oral dose treatment on food metarrestin chow (target dose: 10 mg/kg/day for 10 days).

|  | **Plasma** | **Tumor** | **Spleen** | **Liver** |
| --- | --- | --- | --- | --- |
| AUC_0-24hr_ (ng●hr/mL) | 4160 | 96100 | 109000 | 153000 |
| T_max_ (hr) | 9 | 9 | 15 | 12 |
| C_max_ (ng/mL) | 237 | 5100 | 5840 | 7250 |
| AUC_0-24hr_ Ratio (Tissue/Plasma) |  | 23 | 26 | 37 |
